# Pembrolizumab-Induced Vitiligo in Esophageal Squamous Cell Carcinoma Patient With Durable Complete Response

**DOI:** 10.7759/cureus.19739

**Published:** 2021-11-19

**Authors:** Matthew C Wilkins, Mohamed Elgamal, Igor I Rybkin

**Affiliations:** 1 Hematology and Oncology, Henry Ford Health System, Detroit, USA; 2 Hematology and Medical Oncology, Henry Ford Health System, Detroit, USA

**Keywords:** medical oncology, gastrointestinal oncology, oncology, clinical dermatology, dermatology, vitiligo, pembrolizumab, adverse drug events, esophageal squamous cell carcinoma (scc), cancer immunotherapy

## Abstract

Immune checkpoint inhibitors have emerged as a valuable therapeutic strategy in cancer treatment. Pembrolizumab is an inhibitor of programmed cell death protein 1 (PD-1) and its ligands 1 (PD-L1) and 2 (PD-L2). Disrupting the interaction between PD-L1 expressed on the cancer cell and PD-1 transmembrane protein on immune cells results in reactivation of T cell-mediated cellular immunity. This immune modulation has increased the risk of autoimmune adverse events, which can affect any organ system. Here, we present a case of delayed immune checkpoint inhibitor-induced vitiligo in a 74-year-old female with recurrent metastatic esophageal carcinoma who remains in remission more than five years after initiation of pembrolizumab.

## Introduction

It is estimated that in 2021 there will be 19,260 new cases of esophageal cancer comprising 1% of all new cases of cancer diagnosed in the United States. However, despite medical advances in recent decades, it amounts to 2.6% of all cancer deaths per year. The overall five-year relative survival is 19.9%, improving in earlier stages; however, prognosis remains guarded with a five-year relative survival of localized, regional, and distant disease being 46.4%, 25.6%, and 5.2%, respectively [1].

Several cytotoxic agents are active against esophageal malignancies. Traditional chemotherapy treatments utilize combinations of platinum-based agents, fluoropyrimidines, taxanes, and irinotecan in hopes of increasing response and overall survival rates. Even though these combinations have a better outcome when compared to single-agent use, the durability of response is limited to weeks or months because of their increased risk of treatment-related toxicities [2,3].

Pembrolizumab is a humanized monoclonal immunoglobulin G4 (IgG4) kappa antibody that can reinstate the innate anti-tumor response by blocking the interaction between programmed cell death protein 1 (PD-1) and its ligands 1 (PD-L1) and 2 (PD-L2). PD-1 is a transmembrane receptor expressed on a variety of immune cells, including T cells [4]. The binding of PD-1 to its ligands PD-L1 and PD-L2 results in the transduction of signals that down-regulate T-cell function, reducing the cells’ ability to eliminate neoplastic cells. By expressing PD-L1 on their surfaces, neoplastic cells can co-opt the inhibitory PD-1 signaling pathway and evade T-cell immune surveillance and destruction. Pembrolizumab binds to PD-1 and blocks its binding with PD-L1, removing this cellular “break” on immune activity and restoring anti-tumor response [4].

Cutaneous immune-related adverse events are known complications of immune checkpoint inhibitors with an incidence of pruritus, rash, and vitiligo occurring in 10.6%, 9.31%, and 3.3% of cases, respectively [5]. A previously reported meta-analysis has associated immune checkpoint inhibitor-induced vitiligo with improved outcomes in patients with melanoma [6]. However, immunotherapy-induced vitiligo is rare in patients with non-melanoma malignancies. Subsequently, a few survival analyses have favored longer progression-free survival in patients who developed cutaneous adverse events [6].

## Case presentation

**Figure 1:**
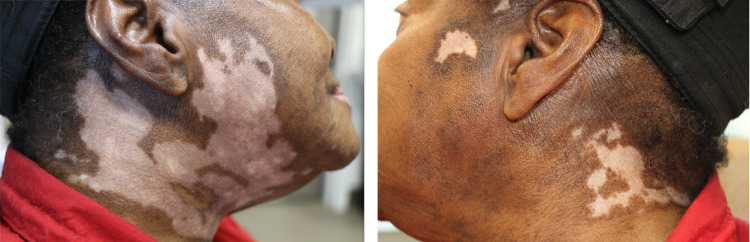
Right and left-sided asymmetric hypopigmented patches of the head and neck.

A 74-year-old female diagnosed with stage IV esophageal squamous cell carcinoma presented to the medical oncology clinic in July 2021 for follow-up 10 months after completing the second course of pembrolizumab. The patient noted progressive asymptomatic hypopigmentation of the head and neck. She denied the use of any new medications including topical ointments, trauma, nevi, melanoma, or history of vitiligo. Physical examination demonstrated well-demarcated, asymmetric, depigmented patches localized to the head and neck (Figure 1). The dermatological evaluation was unremarkable for alternative etiologies. As her manifestations were purely cosmetic, punch biopsy was deferred and concluded to be consistent with immunotherapy-induced vitiligo. No active intervention was required.

**Figure 2:**
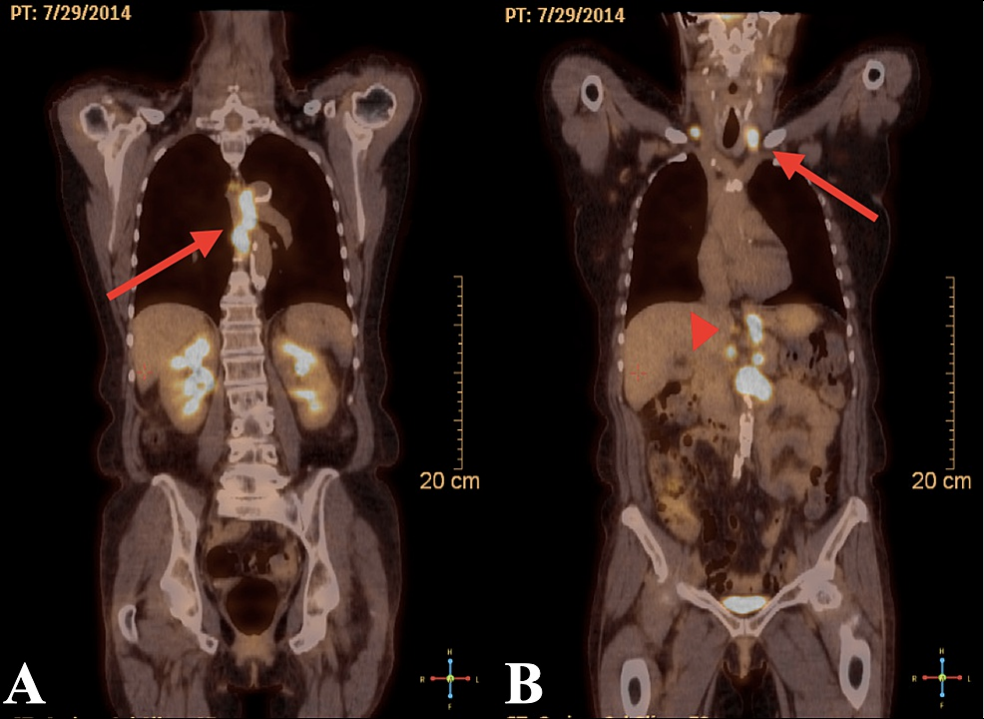
Fluorodeoxyglucose positron emission tomography demonstrating (A) hypermetabolic wall thickening of the mid esophagus consistent with primary malignancy as shown with a red arrow and (B) hypermetabolic bilateral supraclavicular (red arrow) and retrocrural, gastrohepatic, and paraaortic hypermetabolic lymphadenopathy (red arrowhead).

Our patient’s oncological history began in 2014 after presenting with dysphagia and unintentional weight loss. Esophagogastroduodenoscopy (EGD) demonstrated a 5 cm long firm friable esophageal mass at 30 cm from the incisors, Schatzki ring, and erythematous inflammation in the antrum of the stomach. Pathology revealed squamous cell carcinoma, moderately to poorly differentiated. Staging fluorodeoxyglucose positron emission tomography (FDG-PET) showed intense hypermetabolic wall thickening of the mid esophagus with intense hypermetabolic distant metastatic lymphadenopathy consistent with stage IV disease (Figure 2).

**Figure 3:**
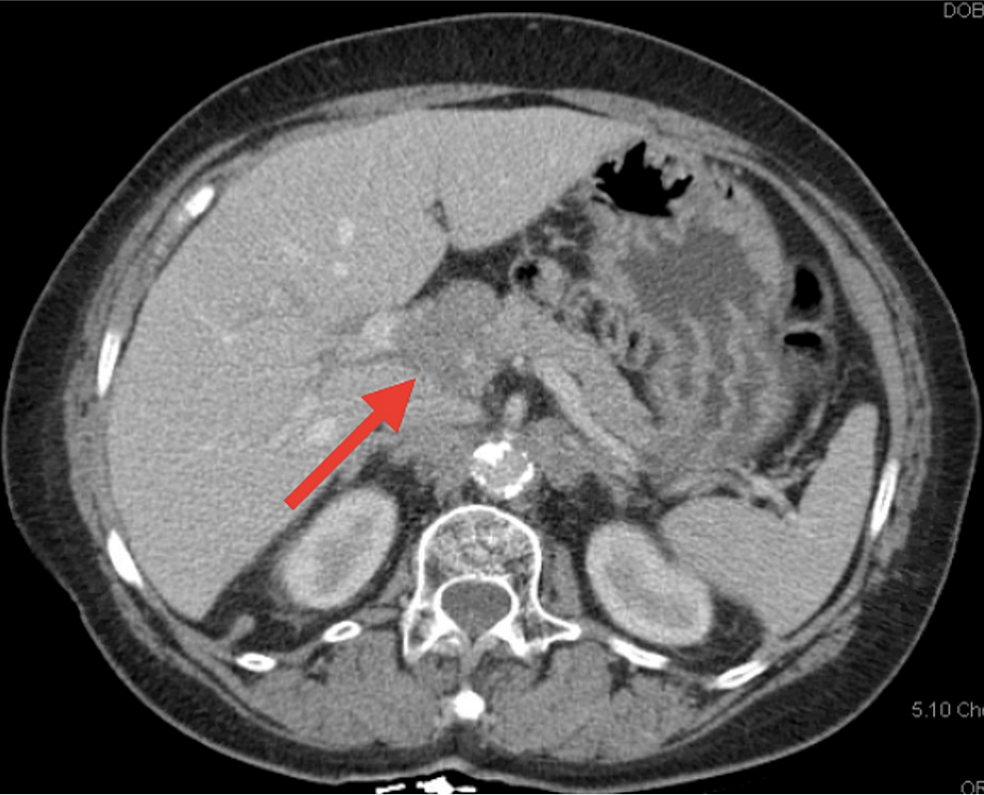
Spiral abdomen and pelvic CT demonstrating enlarging metastatic lymphadenopathy within gastrohepatic ligament shown with a red arrow.

Palliative chemotherapy was initiated with cisplatin 100 mg/m^2^ and fluorouracil 1,000 mg/m^2^/day for six cycles achieving a partial remission until disease progression. She was subsequently treated with second-line fluorouracil 400 mg/m^2^ bolus + 1,000 mg/m^2^/day, irinotecan 180 mg/m^2^, and leucovorin 400 mg/m^2^ (FOLFIRI) achieving another partial remission lasting for almost 11 months. During follow-up, a computed tomography (CT) scan demonstrated enlarging retroperitoneal, para-aortic, caval-atrial, porta hepatis, and gastrohepatic ligament lymph nodes lymphadenopathy (Figure 3) consistent with progressive disease.

**Figure 4:**
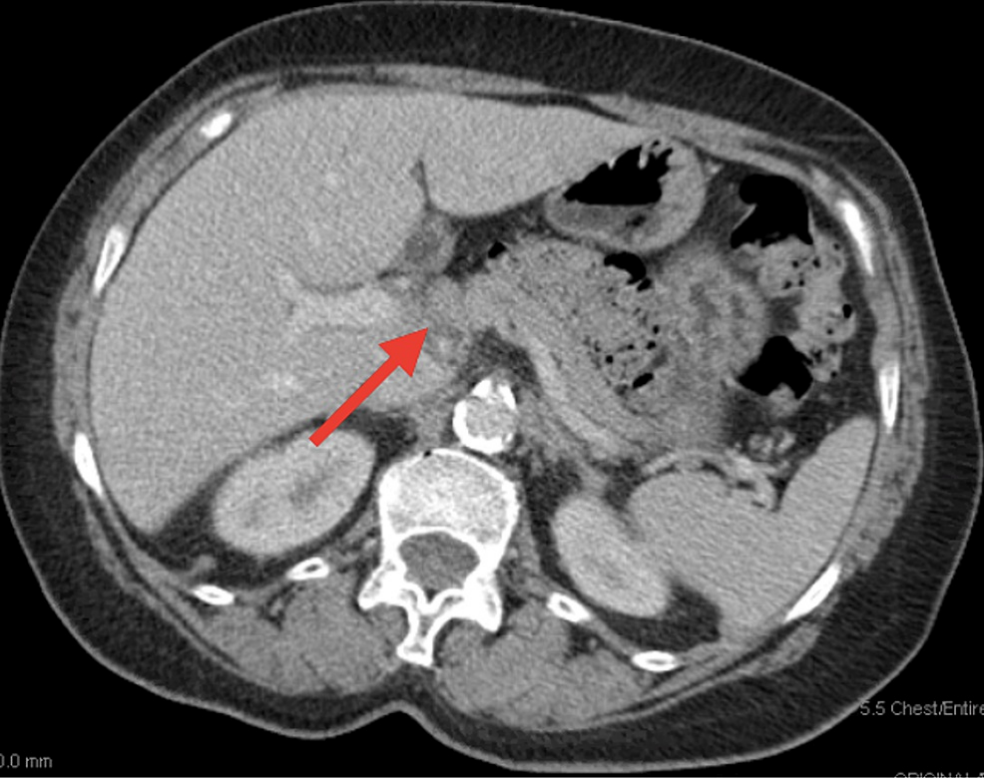
Spiral abdomen and pelvic CT demonstrating decreasing metastatic lymph node within gastrohepatic ligament shown with a red arrow.

In April 2016, the patient was enrolled into clinical trial KEYNOTE-180, a phase II study of pembrolizumab monotherapy in third-line previously treated subjects with advanced/metastatic adenocarcinoma or squamous cell carcinoma of the esophagus or advanced/metastatic Siewert type I adenocarcinoma of the esophagogastric junction. On April 6, 2016, the first dose of pembrolizumab 200 mg was administered and the first interval CT on June 6, 2016 demonstrated interval improvement of mediastinal, upper abdominal, and particularly porta hepatis adenopathy (Figure 4) consistent with response to therapy.

**Figure 5:**
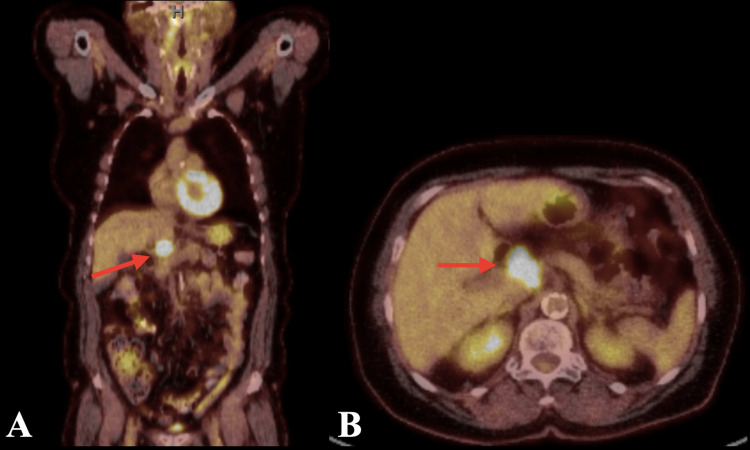
Fluorodeoxyglucose positron emission tomography (A) coronal plane and (B) transverse plane demonstrating hypermetabolic lymphadenopathy within gastrohepatic ligament shown with a red arrow.

The therapy was well tolerated with the only adverse event being grade 2 immunotherapy-induced hypothyroidism in October 2016 after presenting with symptoms of worsening fatigue. Thyroid-stimulating hormone (TSH) confirmed the diagnosis with a level of 150 uIU/mL (reference range: 0.30-5.00 uIU/mL), resolving after the initiation of levothyroxine. Interval imaging was obtained every two to three months per protocol while on therapy and continued to demonstrate disease response eventually achieving complete radiographic remission. Pembrolizumab was continued every three weeks for a total duration of two years with the last dose on March 28, 2018, completing a total of 35 cycles. The patient underwent active surveillance for nine months until January 2019 at which time an FDG-PET scan demonstrated disease recurrence involving the gastrohepatic, paraaortic, and right common iliac lymph nodes (Figure 5).

**Figure 6:**
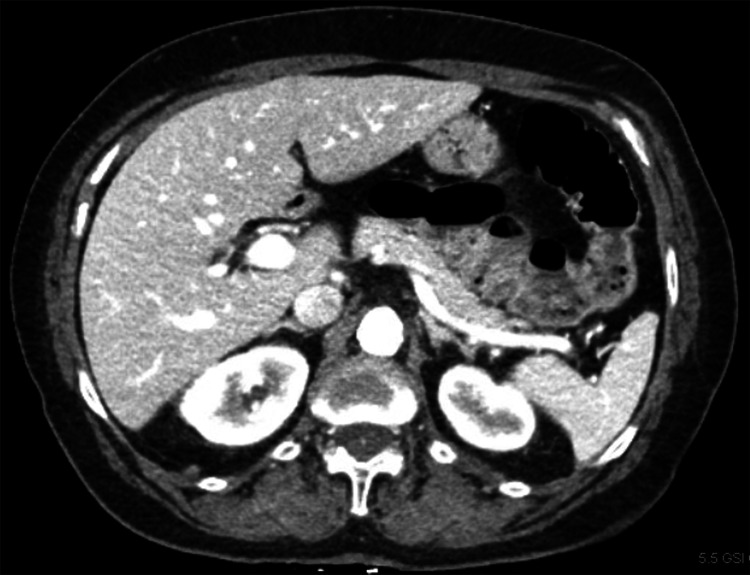
Spiral abdomen and pelvic CT showing complete resolution of the gastrohepatic ligament lymph node.

Pembrolizumab 200 mg every three weeks was re-initiated, completing an additional 35 cycles and achieving a second radiographic complete response per CT imaging obtained in May 2021 (Figure 6).

## Discussion

Pembrolizumab was the first studied immune checkpoint inhibitor in patients with gastroesophageal cancers in the phase Ib KEYNOTE-012 trial in 2016, demonstrating an overall response rate (ORR) of 22% [7]. The FDA granted pembrolizumab approval in 2017, largely based on two notable phase 2 trials KEYNOTE-059 and KEYNOTE-180. Both of these studies demonstrated improved ORR, median progression-free survival (mPFS), and median overall survival (mOS) in previously treated gastroesophageal malignancies treated with single-agent pembrolizumab [8,9]. The open-label, randomized phase 3 KEYNOTE-181 trial went on to compare tumor response to pembrolizumab relative to single-agent chemotherapy in previously treated metastatic esophageal cancer with results suggesting that pembrolizumab significantly improved overall survival versus chemotherapy as second-line therapy for advanced esophageal cancer with PD-L1 positivity/combined positive score (CPS) greater than 10 with a superior safety profile. In CPS > 10 with squamous cell histology, mOS was 10.3 months versus 6.7 months, and in CPS > 10 adenocarcinoma histology, mOS was 6.3 months versus 6.9 months [10]. These data supported the use of incorporating pembrolizumab into the standard of care for esophageal cancer.

Pembrolizumab-induced vitiligo is characterized by less progressive, asymmetric, depigmented white patches on areas with chronic sun exposure. The lesions typically evolve from macules into larger patches [11]. The mechanism of action behind immunotherapy-associated vitiligo is not fully understood in non-melanoma malignancies. In melanoma, it is hypothesized that pembrolizumab activates cytotoxic CD8+ T cells that target melanocyte antigens (gp100, melanoma antigen recognized by T cells [MART1], tyrosinase, or tyrosinase-related proteins) responsible for melanin synthesis [11]. In our patient, we theorize that reactivation of T cell-mediated cellular immunity results in cross-reactivity between normal melanocyte antigens and antigenic epitopes expressed on the tumor cells.

Sanlorenzo et al. reported in a small retrospective study of cutaneous adverse events that the median number of cycles to presentation of immunotherapy-induced vitiligo was eight, ranging from five to 14 cycles; however, it should be noted that this subset population consisted entirely of patients with metastatic melanoma [6]. Though there is a paucity of published data, our patient clearly developed delayed immunotherapy-induced vitiligo in association with disease control, inducing two complete radiographic remissions. The delayed presentation may be a key prognostic factor in maintaining a durable anti-tumor immune response, arguing against the possibility of lead-time bias or time/dose-dependent variable [12].

## Conclusions

Immune checkpoint inhibitors have emerged as a valuable therapeutic strategy in cancer treatment, comprising of a unique toxicity profile when compared to traditional chemotherapy. Cutaneous immune-related adverse events are known complications, commonly presenting with pruritus or rash. Though rare, pembrolizumab-induced vitiligo in patients with non-melanoma malignancies may be indicative of sustained response to immunotherapy and possibly improve overall survival. This proposed association may be related to the number of treatment cycles as well as immunotherapy reinitiation during the treatment course. Further studies are needed to validate this theory in the future.

## References

[1] (2021). Surveillance, Epidemiology, and End Results (SEER) Program. Cancer stat facts: esophageal cancer. https://seer.cancer.gov/statfacts/html/esoph.html.

[2] Sym SJ, Hong J, Park J (2013). A randomized phase II study of biweekly irinotecan monotherapy or a combination of irinotecan plus 5-fluorouracil/leucovorin (mFOLFIRI) in patients with metastatic gastric adenocarcinoma refractory to or progressive after first-line chemotherapy. Cancer Chemother Pharmacol.

[3] Higuchi K, Tanabe S, Shimada K (2014). Biweekly irinotecan plus cisplatin versus irinotecan alone as second-line treatment for advanced gastric cancer: a randomised phase III trial (TCOG GI-0801/BIRIP trial). Eur J Cancer.

[4] Joshi SS, Maron SB, Catenacci DV (2018). Pembrolizumab for treatment of advanced gastric and gastroesophageal junction adenocarcinoma. Future Oncol.

[5] Teulings HE, Limpens J, Jansen SN, Zwinderman AH, Reitsma JB, Spuls PI, Luiten RM (2015). Vitiligo-like depigmentation in patients with stage III-IV melanoma receiving immunotherapy and its association with survival: a systematic review and meta-analysis. J Clin Oncol.

[6] Sanlorenzo M, Vujic I, Daud A (2015). Pembrolizumab cutaneous adverse events and their association with disease progression. JAMA Dermatol.

[7] Muro K, Chung HC, Shankaran V (2016). Pembrolizumab for patients with PD-L1-positive advanced gastric cancer (KEYNOTE-012): a multicentre, open-label, phase 1b trial. Lancet Oncol.

[8] Shah MA, Kojima T, Hochhauser D (2019). Efficacy and safety of pembrolizumab for heavily pretreated patients with advanced, metastatic adenocarcinoma or squamous cell carcinoma of the esophagus: the phase 2 KEYNOTE-180 study. JAMA Oncol.

[9] Fuchs CS, Doi T, Jang RW (2018). Safety and efficacy of pembrolizumab monotherapy in patients with previously treated advanced gastric and gastroesophageal junction cancer: phase 2 clinical KEYNOTE-059 trial. JAMA Oncol.

[10] Kojima T, Muro K, Francois E (2019). Pembrolizumab versus chemotherapy as second-line therapy for advanced esophageal cancer: phase III KEYNOTE-181 study. J Clin Oncol.

[11] Burzi L, Alessandrini AM, Quaglino P, Piraccini BM, Dika E, Ribero S (2021). Cutaneous events associated with immunotherapy of melanoma: a review. J Clin Med.

[12] Hwang SJ, Byth K, Fernandez-Penas P (2015). Time-dependent measurement of adverse events. JAMA Dermatol.

